# Vieussens’ Arterial Ring: Historical Background, Medical Review and Novel Anatomical Classification

**DOI:** 10.7759/cureus.40960

**Published:** 2023-06-26

**Authors:** Konstantinos C Christodoulou, Dimitrios Stakos, Vassiliki Androutsopoulou, Maria Chourmouzi-Papadopoulou, Gregory Tsoucalas, Dimos Karangelis, Aliki Fiska

**Affiliations:** 1 Laboratory of Anatomy, School of Medicine, Democritus University of Thrace, Alexandroupolis, GRC; 2 Department of Cardiac Surgery, Democritus University of Thrace, University Hospital of Alexandroupolis, Alexandroupolis, GRC; 3 Department of Cardiology, Democritus University of Thrace, University Hospital of Alexandroupolis, Alexandroupolis, GRC; 4 Department of General Surgery, NIMTS Medical Institution Military Shareholder Fund, Athens, GRC; 5 Department of History of Medicine and Medical Deontology, School of Medicine, University of Crete, Heraklion, GRC

**Keywords:** raymond de vieussens, anatomical classification, anatomic variant, coronary artery disease, coronary collateral circulation

## Abstract

In coronary artery disease, the presence of Vieussens’ arterial ring (VAR), a ring-shaped anastomosis between the conus branch of the right coronary artery with the left anterior descending artery (LAD), will allow blood flow to return to the obstructed coronary system. We have conducted a literature review, aiming to collect all the existing information about the documented VAR cases and any related pathological conditions. A total of 54 studies entered the review, including 56 patients. The mean age of the patients was 56.12 ± 16.2 years. Angina was present in 53.6% of the patients, with 7.2% of the cases being asymptomatic. Coronary artery disease outweighed (58.9%) as the patients’ most frequent diagnosis. We propose a novel VAR anatomical classification, based on the sites of origin and termination of its course, with six distinct types, for a better understanding and surgical management of VAR. Type IA, originating from the conus branch and terminating in the proximal segment of the LAD was most frequently reported (51.8%). The recognition and the subsequent evaluation of the ring’s anatomy and course are crucial for a customized clinical intervention. When right and left coronary angiographies fail to reveal any collateral circulation, selective conus artery catheterization should be in order. The proposed classification offers a manageable and comprehensive context for the assessment, evaluation and planning of therapeutic strategies of VAR and sets a new terminology frame for treatment guidelines.

## Introduction and background

Understanding the anatomy of the coronary system is significant for clinicians, surgeons and interventional cardiologists [1]. The course, distribution and anatomical relations between coronary arteries are determined antenatally by three embryonic vascular circles: the atrioventricular circle, forming the right coronary artery (RCA) and the left circumflex artery, the inter-ampullary circle which forms the left anterior descending artery (LAD) and the posterior interventricular artery, and the cono/peri-truncal circle which communicates with the truncus arteriosus and anastomoses with the other two vascular circles, completing the arterial blood circulation of the heart [2].

Heart vessels may form clinically significant, homocoronary or intercoronary anastomotic pathways [3]. Their adequacy and extent vary greatly, determining the left ventricular contractility function and the manifestation and consequence of symptoms [4,5]. The collateral pathway between the right and the left coronary circulation is the so-called Vieussens’ arterial ring (VAR) or the arterial circle of Vieussens [6].

It owes its name to the French philosopher, physician and anatomist, Raymond de Vieussens who first described it in 1706. He depicted an epicardial, ring-shaped, vascular structure circling the infundibulum of the right ventricle and connecting the conus branch of the RCA, with an infundibular branch of the LAD or directly with the LAD [7]. VAR provides a life-saving collateral network, which directs blood flow to the right or left arterial system, in cases of coronary artery disease (CAD), total obstruction, or severe stenosis of either RCA or LAD [8]. The origin of the conus branch either from the RCA, or directly from the aorta does not affect the ring’s formation [2].

Collaterals are generally believed to be matured vascular remnants rather than newly formed vessels, and again VAR falls into this category. The embryonic conotruncal circle is located exactly where the VAR is found in adults. Therefore, VAR should be deemed as a persistent conotruncal circle, which serves as an anastomotic vessel in times of coronary artery obstruction [2]. Under normal conditions, the heart vessel network maintains equal pressure on both sides of the VAR, resulting in the absence of blood flow [3]. However, the pressure gradient, due to coronary stenosis or obstruction of proximal segments of RCA or LAD, will cause VAR to progressively dilate, eventually allowing blood flow to the occluded coronary system [9,10].

In this study, we sought to perform an in-depth literature review regarding VAR, aiming (i) to find all the existing information about the documented VAR cases and the diagnostic modalities which can help to unveil them, (ii) to evaluate the symptoms that brought patients to hospital and (iii) to assess pathological conditions related to VAR itself. Finally, we propose a novel VAR classification, to decipher VAR’s presence and set a new terminology frame to facilitate treatment strategies.

Historical background

The majestic French surgeon and anatomist Pierre Dionis (1643-1718) considered his contemporary Raymond de Vieussens (ca. 1633/1641-1715) as one of the greatest anatomists of the French School. His innovative work was celebrated for two centuries in textbooks of anatomy and surgery [11]. Attaining the title Chevalier of France, he became a Counsellor of State and personal Physician to King Lewis XIV. He was a Member of the Royal Academies of both London and Paris and his innovations as a “mature fruit” of an acute and ardent genius, improved internal diseases and surgical anatomy of his era [12].

The information concerning the exact dates of his birth and studies is controversial. Vieussens studied philosophy at Rhodes and medicine at Montpellier and probably at Toulouse in France. He obtained his medical doctorate in 1670 and was appointed as a physician to the hospital of Saint Eloys in Montpellier. During the first decade of his hospital career, he dissected more than 500 cadavers to complete his pioneering study of the central and peripheral nervous systems. Some years later, he shared his practice between the court and anatomy to further improve the depiction of the human body.

**Figure 1:**
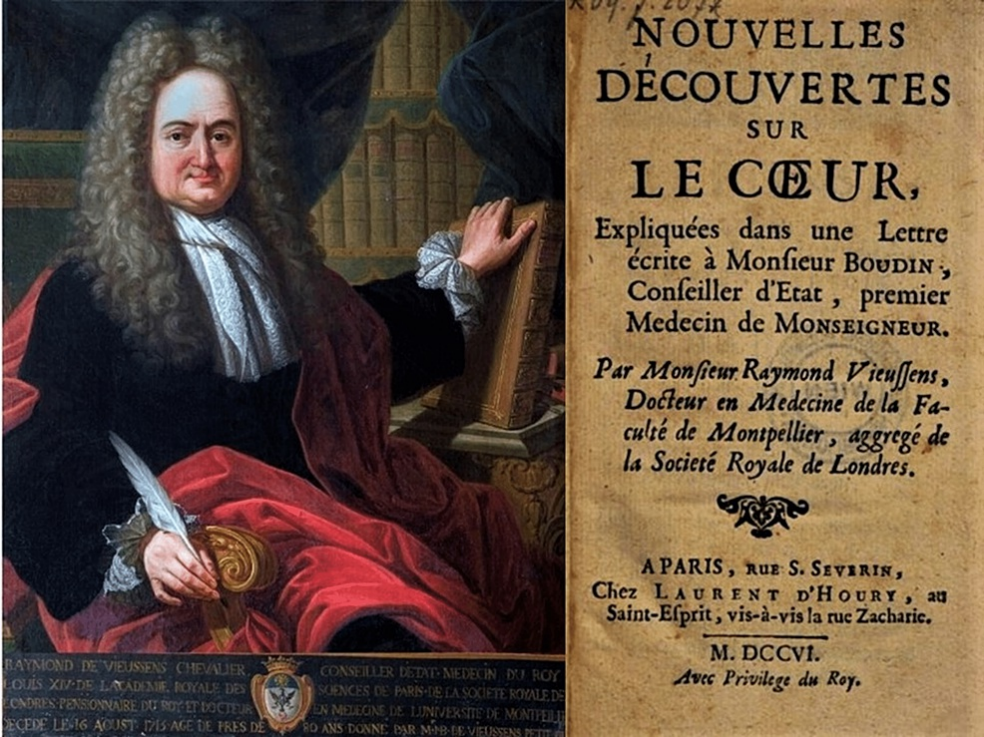
Raymond Vieussens and his work "Traité Nouveau de la Structure et Des Causes Du Mouvement Naturel Du Coeur". Colourized xylography from the first edition of Raymond’s work in Toulouse 1715 titled "Traité Nouveau de la Structure et Des Causes Du Mouvement Naturel Du Coeur" (New Treatise on the Structure and Causes of the Natural Movement of the Heart) published by Jean Guillemette (left side) and his work "Nouvelles Decouvertes Sur Le Coeur" (New Discoveries about the Heart) [7] published by Laurent d'Houry in Paris 1706 (copyright protection has expired).

Vieussens was a prolific writer of treatises in neurology, pathology and anatomy. In 1706 he published his masterpiece titled “Nouvelles Découvertes sur le Coeur” (New Discoveries on the Heart), in which he presented detailed anatomy of the lymphatic and blood vessels of the heart. It is in this original work he recorded, among other abnormalities, the vascular ring named after him, “created by God in his wisdom to ensure the perfect function of human”. Evidence of his inventive approach to heart anatomy is that in order to visualize and map the anatomical effects of cardiac tamponade, mitral stenosis and aortic regurgitation, he ligated the superior and inferior venae cavae and pulmonary veins, and injected saffron dye into the coronary arteries. His studies on the heart were further extended to clinical cardiology in his 1715 publication of “Traité Nouveau de la Structure et Des Causes Du Mouvement Naturel Du Coeur” (New Treatise on the Structure and Causes of the Natural Movement of the Heart). Raymond de Vieussens died on August 16, 1715, almost one year after the death of his greatest and most beloved patron, King Louis XIV. His name endured throughout history, becoming a synonym for the scientific evolution of cardiovascular and nervous system anatomy. It was used to label several anatomical structures, amongst which the celiac ganglia and the innominate cardiac veins, yet it still persists in the name of the coronary collateral ring (Figure 1) [13,14].

## Review

Methods

Search Strategy and Eligibility Criteria

We conducted a PubMed and Scopus database search, using the terms “Vieussens Ring”, “Arterial circle of Vieussens” and “Collateral circulation from the conus artery to the anterior descending artery”. We also searched the Google Scholar database, using the key term “Vieussens arterial ring”. Through the aforementioned search terms, two independent reviewers (KCC and DS) identified eligible articles from inception to March 16, 2021. All articles reporting the presence of the VAR in human subjects were included. Those studies which do not explicitly refer to the VAR, but exhibited its characteristic ring-shaped structure in coronary angiographies were also included. Furthermore, we investigated variations of the conus artery origin, but we excluded any other collateral pathways between the conus artery and the LAD. The inclusion criteria were (i) full-text studies and (ii) English language or official English translation provided. Regarding case-series studies, only articles providing clear documentation about the VAR were included.

Statistical Analysis

All data were recorded in the form of tables, and statistical analysis (pooled analysis) was performed. Continuous variables are reported as mean ± SD and categorical variables as numbers (percentage). Nominal variables were subjected to Fisher’s exact test to evaluate possible associations. Data were analyzed using Statistical Package for the Social Sciences (SPSS Inc., Chicago, IL, USA) for Windows version 10.0 software. A p-value p<0.05 was considered statistically significant.

Results

**Figure 2:**
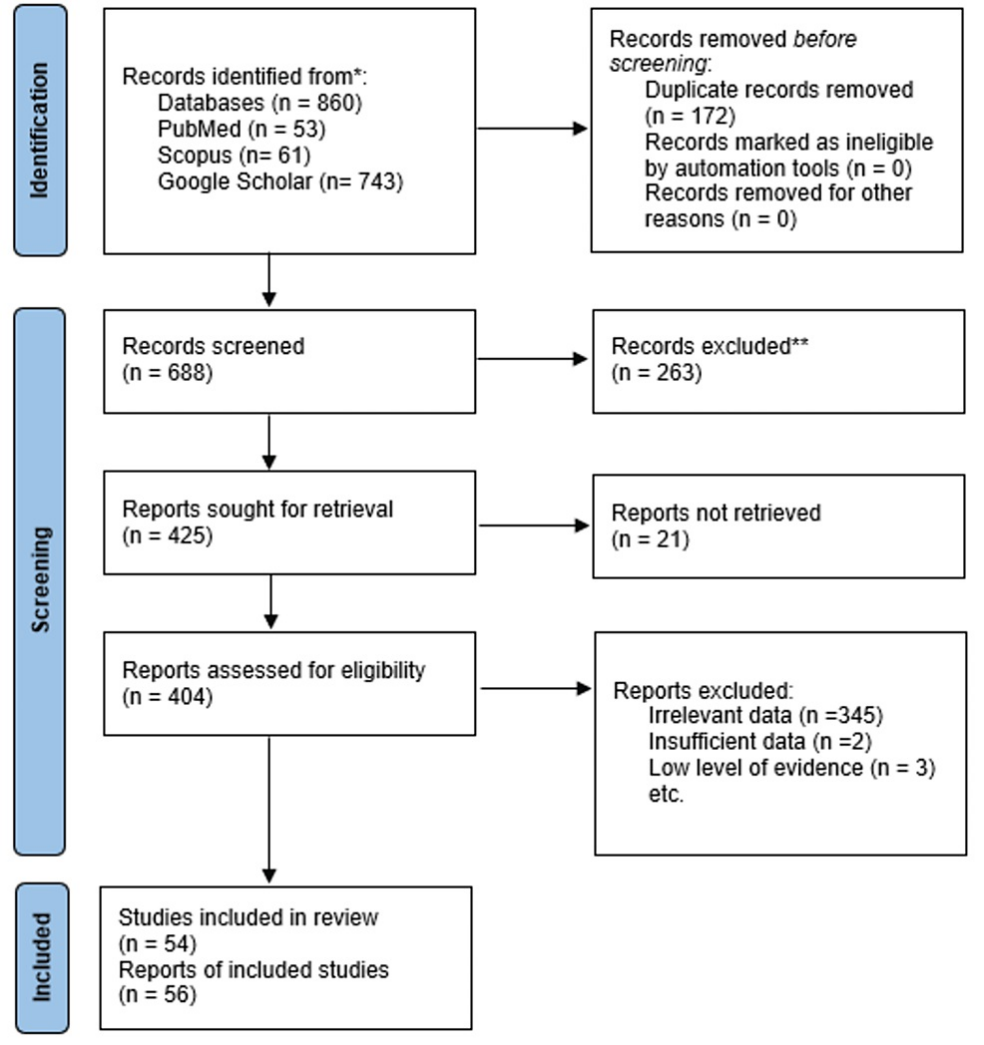
PRISMA flowchart of the article selection process PRISMA = Preferred Reporting Items for Systematic Reviews and Meta-Analyses

The search strategy yielded 688 records after the removal of duplicates. The entire study selection process can be seen in Figure 2.

The current review reports a total of 56 VAR cases, 42 males (75%) and 14 females (25%) (Table 1).

**Table 1:** Summary of the 56 Vieussens’ arterial ring cases, in descending chronological order ALCA = anomalous left coronary artery, ARCAPA = anomalous right coronary artery from pulmonary artery, CA = coronary angiography, CAD = coronary artery disease, CAF = coronary artery fistula, CCTA = coronary computed tomography angiography, ICA = isolated conus artery, LMCA = left main coronary artery, MRI = magnetic resonance imaging, RCO = right coronary ostium, VAR = Vieussens’ arterial ring

Study	No. of cases	Sex	Age	Symptoms	Imaging	ICA	Diagnosis
Musuraca G et al., 2020 [15]	1	Male	71	Dyspnoea	CA, CCTA, Aortography	No	ARCAPA
Tomura N et al., 2020 [16]	1	Male	16	Cardiopulmonary arrest	CA, CCTA	Yes	LMCA atresia
Cimci M et al., 2020 [17]	1	Male	67	Dyspnoea	CA	Yes	CAD
Cellina M et al., 2020 [6]	1	Male	61	Angina	CCTA	No	CAF
Christodoulou KC et al., 2020 [18]	1	Male	58	Angina, Dyspnoea	CA	Yes	CAD
Roik D et al., 2020 [19]	1	Male	1	Heart failure	CCTA	No	ALCA
Sinha M et al., 2020 [20]	1	Male	29	Angina	CCTA	Yes	LMCA atresia
Pandey NN et al., 2019 [21]	1	Female	28	Dyspnoea	CCTA	No	RCO atresia
Lee SH et al., 2019 [22]	1	Male	55	Fever, headache	Transthoracic, Transesophageal echocardiography, CCTA	No	Infective endocarditis, VAR Aneurysm, CAF
Malik SA et al., 2019 [23]	1	Male	59	Angina	CA	No	CAD
Alsancak Y et al., 2018 [24]	1	Female	57	Angina	CA	No	CAF
Brolund-Napier C et al., 2017 [25]	1	Male	65	Chest discomfort, dyspnoea	CA, CCTA	No	CAD, VAR Aneurysm, CAF
De Cecco CN et al., 2017 [26]	1	Male	53	Asymptomatic	CCTA	No	VAR Aneurysm
Poulidakis E et al., 2016 [27]	1	Male	55	Angina	CA	No	CAD
Ni J et al., 2016 [28]	1	Female	56	Dyspnoea	CCTA	No	VAR Aneurysm, CAF
Plácido R et al., 2016 [4]	1	Male	76	Angina, Ischaemic stroke	CA, CCTA, Aortography	Yes	LMCA atresia, CAD
Unzué L et al., 2015 [29]	1	Male	67	Dyspnoea	CA	No	LMCA atresia
Srikumar S et al., 2015 [30]	1	Female	45	Angina	CA	Yes	CAD
Calvillo-Batllés P et al., 2015 [31]	1	Female	65	Asymptomatic	Transesophageal echocardiography, CA, CCTA, MRI	Yes	VAR Aneurysm, CAF
Alhejily WA et al., 2015 [32]	1	Female	66	Angina	CA	No	CAD
Ramsdale KA et al., 2015 [33]	1	Male	70	Angina	CA	No	CAD
Patel H et al., 2015 [34]	1	Female	86	Fatigue, diarrhea, epigastric pain	CA	No	CAD
Alsancak Y et al., 2015 [35]	1	Male	58	Angina	CA, CCTA	No	VAR Aneurysm, CAF
Feng J et al., 2014 [36]	1	Female	60	Chest discomfort	CCTA	Yes	VAR Aneurysm, CAF
Lee HY et al., 2014 [37]	1	Male	53	Chest discomfort, palpitations	Transthoracic echocardiography, CA, CCTA	Yes	Hypoplastic right coronary artery system, VAR Aneurysm, CAF
Deora S et al., 2014 [8]	1	Male	66	Angina	CA, CCTA	Yes	CAD
Bamoshmoosh M et al., 2013 [38]	1	Male	65	Angina, Dyspnoea	CCTA	No	CAD
Singla R et al., 2013 [39]	1	Male	56	Angina	CA	Yes	CAD
Yadav A et al., 2013 [40]	1	Male	62	Jaw pain	CA, CCTA	No	LMCA atresia, CAD
Deng B et al., 2013 [41]	1	Female	77	Lower-extremity oedema, dyspnoea	Chest x-ray, Transthoracic echocardiography, CCTA	No	VAR Aneurysm
Cam F et al., 2013 [5]	1	Male	54	Myocardial infarction	CA	Yes	CAD
Erbas G et al., 2011 [42]	1	Male	44	Angina	CA, CCTA	No	Hypoplastic right coronary artery system, CAD, CAF
Saremi F et al., 2011 [43]	1	Male	46	Palpitations, lightheadedness	CA, CCTA	No	RCO atresia
Dhanoa D et al., 2011 [44]	1	Male	65	Angina	CA, CCTA	No	LMCA atresia
Atallah PC et al., 2011 [45]	1	Male	61	Palpitations, dyspnoea, chest discomfort	CA, CCTA	Yes	Hypoplastic left coronary artery system, CAD
Hirzallah MI et al., 2010 [46]	1	Female	45	Presyncope, palpitations, dyspnoea	Transthoracic echocardiography, CA, CCTA	No	VAR Aneurysm, CAF
de Agustín JA et al., 2010 [47]	1	Male	54	Angina	CA, CCTA	Yes	CAD
	1	Male	74	Angina	CA, CCTA	Yes	CAD
	1	Male	60	Angina	CA, CCTA	No	CAD
Wynn GJ et al., 2010 [48]	1	Male	60	Angina	CA	No	CAD
Owen AR et al., 2009 [49]	1	Female	67	Syncopal episode	CT pulmonary angiography, CT aortography	No	VAR Aneurysm
Díaz-Zamudio M et al., 2009 [50]	1	Female	42	Angina	CCTA	No	CAD
Chan CY et al., 2009 [10]	1	Female	73	Asymptomatic	Chest x-ray, Transthoracic echocardiography, CA, CCTA	No	CAD, VAR Aneurysm, CAF
de Agustin JA et al., 2009 [51]	1	Male	71	Angina	CA, CCTA	Yes	CAD
Tsiamis E et al., 2008 [52]	1	Male	56	Angina	CA	Yes	CAD
Baskurt M et al., 2008 [53]	1	Male	57	Angina	CA	Yes	CAD
Chou M et al., 2007 [54]	1	Male	50	Angina	CA, CCTA	No	LMCA atresia
Gupta V et al., 2007 [55]	1	Male	52	Angina	CA	No	CAD, VAR Aneurysm, CAF
Hansen MW et al., 2006 [56]	1	Male	58	Asymptomatic	CCTA, MRI	No	CAD
Shen AY et al., 2006 [57]	1	Male	58	Angina	Transthoracic echocardiography, CA	No	CAD
Funabashi N et al., 2005 [58]	1	Male	70	Angina	CA, CCTA	No	CAD
Kocica MJ et al., 2004 [59]	1	Female	51	Chest discomfort	Chest x-ray, Transthoracic, Transesophageal echocardiography, CA	Yes	VAR Pseudoaneurysm
Germing A et al., 2003 [60]	1	Male	39	Angina	CA	No	CAD
O'Leary EL et al., 1998 [61]	1	Male	63	Bilateral lower-extremity oedema, Weight gain, dyspnoea	CA	No	CAD, Heart failure
Feld S et al., 1995 [62]	1	Male	67	Angina	CA	Yes	CAD
Chiu IS et al., 1994 [63]	1	Male	3	Tachypnea	CA, Aortography	No	Criss-cross heart

**Figure 3:**
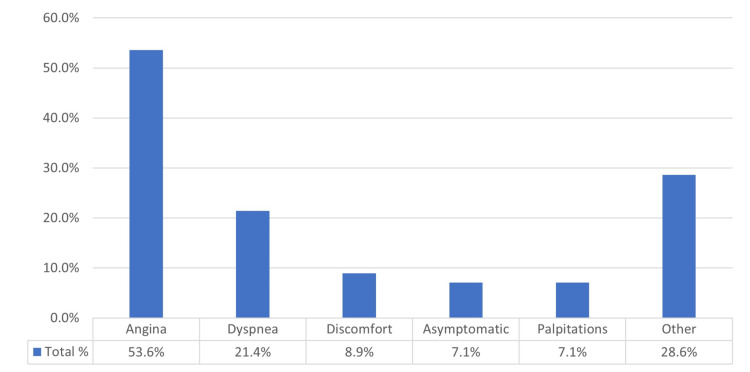
Distribution of the symptoms resulted in hospital admission Most reported symptoms related to Vieussens’ arterial ring.

**Figure 4:**
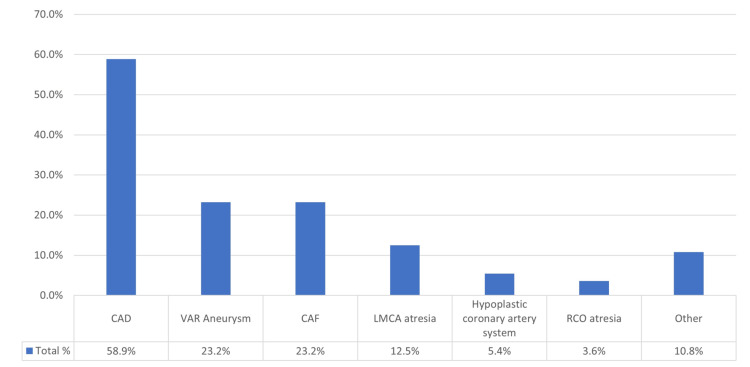
Summary of diagnoses made in the 56 Vieussens’ arterial ring cases Diagnosis distribution of the reviewed cases. CAD = coronary artery disease, CAF = coronary artery fistula, LMCA = left main coronary artery, RCO = right coronary ostium, VAR = Vieussens’ arterial ring

The mean age of patients was 56 years old (range 1-86 years). All cases were adults over 28 years of age, apart from three studies reporting the cases of a 16-year-old adolescent, a three-year-old child and a 10-week-old baby, respectively. Twenty per cent of the patients were smokers, while dyslipidemia, hypertension and diabetes were present in 16%, 21.5% and 12.5% of the study cohort, respectively. Approximately 16% of the patients had a previous myocardial infarction and/or a family history of CAD, while 15 patients (26.8%) exhibited an unremarkable past medical history. Multifactorial symptomatology resulting in hospital admission clustered into patients as exhibited in Figure 3. Angina was the leading symptom, present in 30 out of 56 patients (53.6%). However, we noted four asymptomatic cases (7.2%), in which VAR was an incidental radiological finding. In all cases, VAR presence was confirmed by radiological examination, most commonly by coronary angiography (43 patients, 76.8%) and computed tomography (CT) coronary angiography (33 patients, 58.9%). The conus artery was found to have a separate origin from the right sinus of Valsalva in 20 cases (35.7%). Concerning patients’ diagnosis, CAD outweighed, as it was found in 33 out of 56 patients (58.9%); 17 had multivessel CAD (30.5) and six patients (10.7%) had triple vessel CAD (Figure 4).

VAR-related pathologies, such as abnormal communications with the main pulmonary artery and aneurysmal dilations, were observed in 16 cases (28,6%). More than half of the women (57.1%) (p = 0.014) presented with VAR-related pathology. Both coronary artery fistula (CAF) and VAR aneurysm were recorded in 13 cases (23.2%), either simultaneously or independently of one another. The largest aneurysm measured 6.5*6 cm. Additionally, there was one study reporting the first-ever presence of a gigantic VAR pseudoaneurysm, in a 51-year-old female patient, which was measured in volume 4*5*4 cm.

Discussion

The conus branch of the RCA passes anteriorly and upward around the pulmonary infundibulum where it terminates with several branches [61]. It may mature into such a well-formed vessel, as to provide an extensive arterial distribution to the anterior ventricular wall [14]. It may have a separate origin in the right sinus of Valsalva in approximately 40% of the population. When such an anatomical variation exists, the conus branch is referred to as the “isolated conus artery” (ICA) or “the third coronary” [39,47]. Accordingly, the left infundibular branch, which is the first ventricular vessel originating from the LAD, when present, it passes anteriorly around the arterial conus [59]. Often, these two vessels anastomose, forming a collateral arterial system (VAR) that connects the RCA with the LAD [2].

Aetiology

VAR does not form only due to CAD; it could also develop as a result of congenitally hypoplastic CAD [45]. Seven cases reported the VAR in association with left main coronary artery (LMCA) atresia [4,16,20,29,40,44,54], two cases with right coronary ostium (RCO) atresia [21,43], and three studies in conjunction with a hypoplastic left or right coronary arterial system [37,42,45].

In a reported case of a right dominant circulation along with the absence of both the ostium and the LMCA, the patient survived through a VAR: a large winding conus artery, originating directly from the right sinus of Valsalva, which provided blood supply to the LAD and circumflex artery junction [20]. Plácido et al. highlighted the significance of VAR in the first documented case of a concomitant presence of LMCA atresia and a severely occluded RCA. In their study, the left ventricular ejection fraction was sustained by a collateral network from the conus branch to the mid-portion of LAD [4].

Atallah et al. placed emphasis on the existence of VAR in a case of innate hypoplasia of the entire left arterial circulation [45], with an anastomotic network between the isolated conus artery and the LAD providing adequate arterial flow. In all these three cases, the major factor triggering the ring’s emergence was the immaturity or absence of the left coronary vessels, rather than obstructive lesions. Notwithstanding the two distinct aetiologies, the pathophysiologic mechanism of the ring’s formation is presumed the same: the pressure gradient [45].

Symptoms

Most of the studied cases (58.9%) reported CAD signs, so angina was justifiably the most common symptom. Out of the 30 cases presented with angina 24 (80%) were associated with CAD or with VAR aneurysm (p = 0.003). For the classification of the cluster of symptoms related to angina, this study was based on Ferry’s and Greenslade’s works [64,65]. Symptoms like chest pressure and chest pain fell into the “angina” category, thus explaining its high percentage. On the other hand, “chest discomfort” was classified as a distinct clinical entity, as we consider that it describes the absence of actual cardiac/chest pain [64,65].

The VAR may rarely be accompanied by vascular pathology [46]. CAFs comprise 14% of all coronary anomalies recorded in the literature and are found in approximately 0.002% of the general population [66]. A particular type of CAF appears when tortuous VAR vessels carrying blood from the RCA and the LAD drain into the main pulmonary artery. The fistula’s size and its secondary hemodynamic changes account for its clinical manifestations, varying from none to heart failure [67]. Gupta and colleagues were the first to describe the existence of a left-to-right shunt, due to the VAR-fistula, triggering the "coronary steal phenomenon” and leading to symptoms of myocardial ischaemia [55]. According to Chenjin Ge et al., the most frequent symptoms in patients with VAR fistulas were chest pain and dyspnoea [67]. We found that of the 13 CAF cases reported in the literature, five patients presented with angina [6,24,35,42,55], three with chest discomfort [25,36,37] and three with dyspnoea [25,28,46], whereas two were asymptomatic [10,31].

Supplementary, two cases were noted of a CAF with a hypoplastic right coronary arterial system [37,42]. Lee and colleagues described the case of a 55-year-old male with infective endocarditis and severe mitral regurgitation, suggesting that in the absence of any other obvious causes, such conditions should be attributed to CAFs [37]. Sometimes, the part of the ring proximal to the fistula becomes dilated leading to aneurysm formation, plus promoting premature coronary atherosclerosis or even cardiac arrhythmia and heart failure [6].

VAR aneurysm is a unique type of arterial dilation located in the intercoronary pathway between the conus artery and the LAD [67]. Theories about the pathogenesis of VAR aneurysms do not exist, although there are several proposed mechanisms for the development of coronary artery aneurysms in general. Probably, the pressure gradient, which is responsible for the VAR dilation, is the precipitating factor for the formation of VAR aneurysms. The present review includes 13 VAR aneurysm cases, three solitary [26,41,49] and 10 in concurrence with CAFs [10,22,25,28,31,35-37,46,55]. With respect to the study cohort, 43% of the women (p = 0.011) presented with an aneurysm, compared to 14% of the men. Dyspnoea was the most common symptom, having been reported in four studies [25,28,41,46], while three patients were completely asymptomatic [10,26,31]. Owen and co-authors described the first ever documented case of a ruptured VAR aneurysm in a 67-year-old female. In their study, the patient presented with hemopericardium and cardiac tamponade, requiring emergency surgery [49]. Deng et al. documented a similar case of a clinically acute presentation of a VAR aneurysm, accompanied by hemopericardium and pericardial adhesions, probably due to tuberculosis, while the aneurysmal sac was intact [41].

Diagnosis

In all cases, radiology certified the diagnosis of VAR. Coronary angiography is the “gold standard” imaging modality when the evaluation of the coronary arteries is in order [38]. However, the multidetector CT complements or even competes against the established, yet invasive coronary angiography [9], as it can precisely provide an in-depth depiction of VAR origin, course, dimensions, drainage site and anatomic relations with adjacent structures [6].

Anatomical Classification

VAR’s clinical significance depends on its anatomic course on the heart’s anterior surface, as it is determined from its origin, either from the conus branch or the ICA, and its site of drainage to the LAD (proximal, medial, and distal segment) [68].

KCC and DS thoroughly reviewed the given coronary angiographies, CT coronary angiographies and MRIs in every study, in order to collect all the available information regarding the course of the ring, along with any accompanying pathology. Hence, we propose a functional and easy-to-apply VAR classification. According to this, VAR is first designated as type I or II depending on its origin from the conus branch or directly from the right sinus of Valsalva, respectively. Additionally, the ring is coded with an A, B or C according to its termination in the proximal, medial or distal segment of the LAD, respectively. Ultimately, six types of VAR are formed (Table 2). Any VAR type may be accompanied by vascular pathology (aneurysm and/or CAF).

**Table 2:** Proposed anatomical classification of Vieussens’ arterial ring ICA = isolated conus artery, LAD = left anterior descending artery

		Origin	
		I (conus branch)	II (ICA)
Segment of LAD	A (Proximal)	IA	IIA
	B (Medial)	IB	IIB
	C (Distal)	IC	IIC

Evidently, in the case of RCA occlusion, the origin and the terminal point of the vessel’s blood flow reverse, drifting by the pressure gradient. However, as our classification focuses solely on the ring’s epicardial course, this shift does not modify any of the six VAR variants. In addition, coronary anomalies such as LMCA and RCO atresia [4,16,20,21,29,40,43,44,54], hypoplastic left or right coronary artery system [37,42,45], an anomalous right coronary artery from the pulmonary artery (ARCAPA) [15] and anomalous left coronary artery (ALCA) [19] do not seem to further differentiate the VAR’s formation. Thus, they fall within any of the proposed six VAR types.

It seems that VAR more commonly originates from the conus branch and terminates to the proximal segment of the LAD, since more than half of the 56 cases illustrated in the review, 29 cases (51.8%) were classified as type IA [6,15,19,21-26,28,30,32,33,35,40-44,46-49,54-56,60,61,63]. Type IIA follows with 10 cases (17.9%) [16,18,20,31,36,37,39,53,59,62], and types IB [10,29,38,27,34,50,57] and IIB [4,5,8,47,45,51] were found in seven cases each (12.5%). Type IIC was present in two studies (3.6%) [17,52] and type IC was the rarest, found in a single study (1.8%) [58].

Doğan et al. suggested a different classification system, predominantly based on the presence of a coronary anomaly such as an aneurysm, fistula or single coronary artery [9]. Although this categorization is helpful, our proposed classification offers a more comprehensive evaluation of the ring’s anatomy, critical for the clinical decision-making process especially when percutaneous coronary intervention is planned.

Clinical Implications

After total occlusion of a coronary artery, the development of a collateral network through the septal, branch or epicardial collaterals can provide perfusion to the affected cardiac segment, corresponding to 70% of the basal conditions, thus precluding myocardial necrosis and preserving left ventricular contractility until adequate reperfusion (stenting or Bypass) [69]. The auto-revascularization provided by the collaterals is sometimes sufficient to even obviate the necessity of operation. Furthermore, the spontaneous thrombosis of the collaterals is less likely to occur, compared to a saphenous vein graft [3]. Consequently, VAR can be the source of various anastomotic pathways, relieving angina and allowing patients to survive even the most severe myocardial infarctions [2]. A striking example is the survival of patients with bilateral occlusion of RCA and LMCA [23].

**Figure 5:**
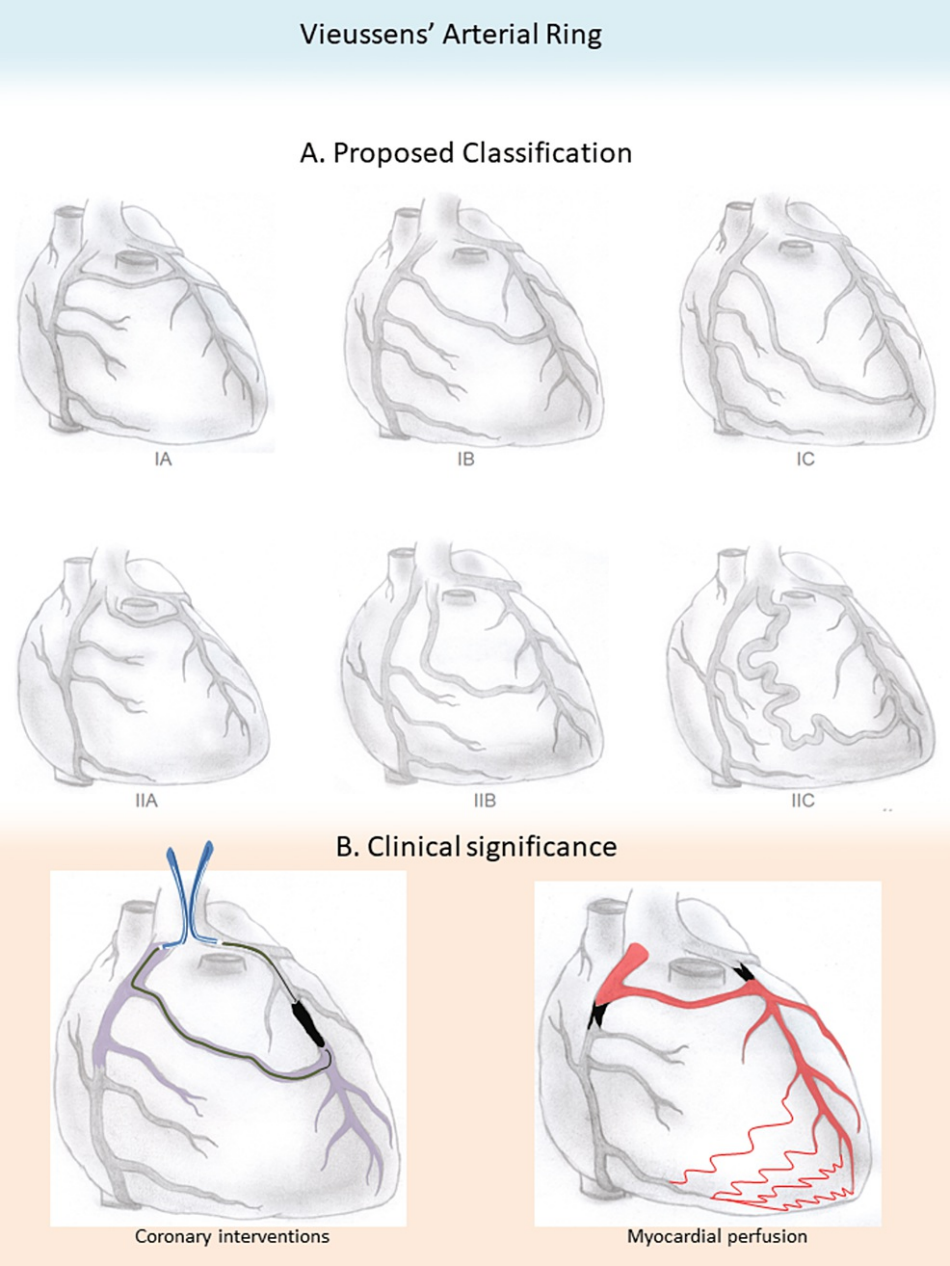
Vieussens’ arterial ring; Proposed anatomical classification and clinical significance. (A) The ring’s anatomic course is determined from its origin, either from the conus branch or from the isolated conus artery and its site of drainage to the LAD, either proximal, medial or distal. Accordingly, six types of VAR are formed, with type IA being the most common one. (B) In coronary artery disease of either RCA or LAD, VAR can naturally bypass the obstructions, providing a life-saving collateral network towards the occluded coronary system. Also, its use during the percutaneous intervention can confirm the intraluminal wire position after crossing and provide an alternative to the anterograde approach in case of crossing failure. LAD = left anterior descending artery, RCA = right coronary artery, VAR = Vieussens’ arterial ring

**Figure 6:**
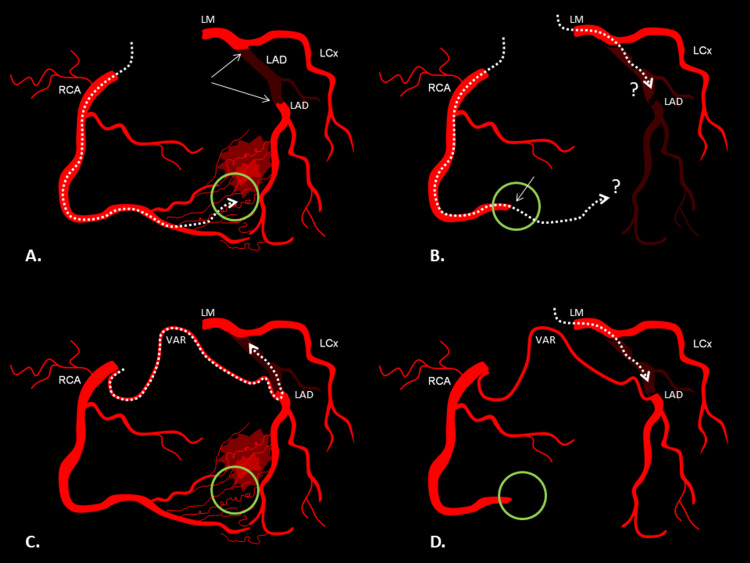
A possible role of Vieussens’ arterial ring (VAR) in coronary interventions (A) In the absence of VAR, a retrograde approach for a totally occluded (arrows) left anterior descending (LAD) artery may fail because of tortuous, perforated (red haziness) or non-interventional collaterals (green circle). (B) Similarly, in the absence of VAR, both retrograde and antegrade approaches may fail in cases of coexisting distal right coronary artery (RCA) occlusion (green circle, arrow) thus precluding collateral formation and distal LAD visualization for safe guidewire advancement. (C) On the contrary, when VAR is present, it can be used as a path for retrograde guidewire advancement to occluded LAD or (D) for LAD visualization in the antegrade attempt in case of coexisting distal RCA occlusion. dotted line = guidewire; LM = left main; LCx = left circumflex artery

In the past, an extended collateral network was the main argument against chronic total occlusion revascularization [70]. Nowadays, it is widely accepted that revascularization, significantly improves patients’ prognosis irrespective of collaterals, although their existence increases the effectiveness of percutaneous coronary intervention [71]. The failure of right and left coronary angiography to reveal any collaterals towards an occluded LAD or RCA, should always be an indication for selective conus artery catheterization [72]. Sometimes the only source of collateral circulation is the ICA, therefore its cannulation, although potentially arrhythmogenic, may affect the patient’s prognosis [39]. The simultaneous contralateral injection of the conus branch, during percutaneous intervention of an occluded LAD, will “reveal” the direction of the distal target vessel, confirming the intraluminal wire position after crossing. Furthermore, in the event of crossing failure, a well-formed conus artery may provide an alternative to the anterograde approach (Figure 5 and Figure 6) [72].

The presence of VAR in multi-vessel CAD does not affect surgical planning regarding myocardial reperfusion [73]. However, it is of the utmost importance for the surgeon to know the ring’s exact course, so as to avoid damaging it during right infundibulum manipulations [47]. Coronary artery bypass surgery with poor collateral connections presents an increased risk for postoperative complications such as stroke and death. In contrast, patients with robust collaterals have improved post-operative survival [73]. Overall, the extended network of collateral connections has a protective role regarding perioperative myocardial infarction during off-pump coronary artery bypass surgery and is associated with improved one-year event-free survival [74].

Limitations

Despite the extensive literature research, the number of studies and cases eventually included in the article is limited. Therefore, the small sample size and the numerical difference between males and females may lead to disputable results.

## Conclusions

VAR represents a unique form of coronary collateral circulation. In acute coronary syndromes, this life-saving vessel potentially bypasses the obstruction, preserving the left ventricle function and performance. In addition, the existence of VAR is associated with congenital coronary anomalies. Regardless of the clinical entities, it is presumed that the triggering factor leading to the VAR’s formation is the developing pressure gradient, between two sides of the coronary circulation.

The recognition and the subsequent evaluation of the ring’s anatomy are of the utmost importance for a personalised clinical intervention. The proposed classification will optimize the assessment and evaluation of VAR and will facilitate planning for revascularization strategies.
